# Accuracy of 2 Rapid Antigen Tests During 3 Phases of SARS-CoV-2 Variants

**DOI:** 10.1001/jamanetworkopen.2022.28143

**Published:** 2022-08-24

**Authors:** Paul K. Drain, Meagan Bemer, Jennifer F. Morton, Ronit Dalmat, Hussein Abdille, Katherine K. Thomas, Timsy K. Uppal, Derrick Hau, Heather R. Green, Marcellene A. Gates-Hollingsworth, David P. AuCoin, Subhash C. Verma

**Affiliations:** 1 Department of Global Health, University of Washington, Seattle; 2 Department of Medicine, University of Washington, Seattle; 3 Department of Epidemiology, University of Washington, Seattle; 4 Department of Microbiology and Immunology, School of Medicine, University of Nevada, Reno

## Abstract

**Importance:**

Variants of SARS-CoV-2 have sequence variations in the viral genome that may alter the accuracy of rapid diagnostic tests.

**Objective:**

To assess the analytical and clinical accuracy of 2 rapid diagnostic tests for detecting SARS-CoV-2 during 3 phases of variants.

**Design, Setting, and Participants:**

This diagnostic study included participants aged 18 years or older who reported onset of COVID-19–like symptoms within the prior 5 days and were tested at multiple COVID-19 testing locations in King County, Washington, from February 17, 2021, to January 11, 2022, during 3 distinct phases of SARS-CoV-2 infection (pre-Delta, Delta, and Omicron).

**Interventions:**

Two anterior nasal swab specimens were collected from each participant—1 for onsite testing by the SCoV-2 Ag *Detect* Rapid Self-Test and 1 for reverse transcriptase–polymerase chain reaction (RT-PCR) testing.

**Main Outcomes and Measures:**

The analytical limit of detection of the 2 rapid diagnostic tests (SCoV-2 Ag *Detect* Rapid Self-Test and BinaxNOW COVID-19 Ag Card) was assessed using Omicron (B.1.1.529/BA.1), Delta (B.1.617.2), and a wild-type (USA-WA1/2020) variant. Diagnostic sensitivity and specificity of clinical testing for the rapid antigen tests were compared with that of RT-PCR testing.

**Results:**

A total of 802 participants were enrolled (mean [SD] age, 37.3 [13.3] years; 467 [58.2%] female), 424 (52.9%) of whom had not received COVID-19 vaccination and presented a median of 2 days (IQR, 1-3 days) from symptom onset. Overall, no significant differences were found in the analytical limit of detection or clinical diagnostic accuracy of rapid antigen testing across SARS-CoV-2 variants. The estimated limit of detection for both rapid nucleocapsid antigen tests was at or below a 50% tissue culture infectious dose of 62.5, and the positive percent agreement of the SCoV-2 Ag *Detect* Rapid Self-Test ranged from 81.2% (95% CI, 69.5%-89.9%) to 90.7% (95% CI, 77.9%-97.4%) across the 3 phases of variants. The diagnostic sensitivity increased for nasal swabs with a lower cycle threshold by RT-PCR, which correlates with a higher viral load.

**Conclusions and Relevance:**

In this diagnostic study, analytical and clinical performance data demonstrated accuracy of 2 rapid antigen tests among adults with COVID-19 symptoms across 3 phases of SARS-CoV-2 variants. The findings suggest that home-based rapid antigen testing programs may be an important intervention to reduce global SARS-CoV-2 transmission.

## Key Points

**Question:**

Are rapid antigen tests analytically and clinically accurate for detecting variants of SARS-CoV-2?

**Findings:**

In this diagnostic study of 802 adults reporting COVID-19–like symptoms within the prior 5 days, no significant differences were found in the analytical limit of detection or clinical diagnostic accuracy of 2 rapid antigen tests across 3 epidemic phases of SARS-CoV-2 variants. The positive percent agreement ranged from 81% to 91% across the 3 phases of variants.

**Meaning:**

This study found that 2 rapid antigen tests had consistent analytical and clinical accuracy across 3 phases of circulating SARS-CoV-2 variants.

## Teaser

This diagnostic study assesses the analytical and clinical accuracy of 2 rapid antigen tests in detecting 3 variants of SARS-CoV-2 among adults with symptoms of COVID-19.

## Introduction

More than 500 million cases of confirmed SARS-CoV-2 infection and 6.2 million deaths from COVID-19 have been reported to the World Health Organization,^1^ and these numbers may be vastly underestimated.^2,3^ Implementation of diagnostic testing for acute SARS-CoV-2 infection has been essential to identify COVID-19 cases, reduce transmission, and inform public health measures.^4,5^ Initially, SARS-CoV-2 testing relied on laboratory-based reverse transcriptase–polymerase chain reaction (RT-PCR) of nasopharyngeal specimens.^6^ Although results of nucleic acid amplification tests have been critical, they have remained expensive and technically and logistically challenging.^7,8^

Rapid diagnostic tests can be used to diagnose acute SARS-CoV-2 infection.^9,10,11^ However, antigen-based assays that rely on antibody conjugation for capture and detection of viral proteins have not been universally endorsed for diagnostic testing.^12,13,14^ Rapid antigen–based diagnostic tests have now become widely abundant^15^ and may be useful to facilitate testing in community- and home-based settings, expedite treatment initiation, and optimize isolation periods.^5,8,16,17,18^

Variants of SARS-CoV-2 have sequence variations in the viral genome that may alter the accuracy of rapid diagnostic tests.^5^ Molecular tests can be affected by single-point sequence variations, whereas antigen tests may require multiple sequence variations to change the confirmation of viral protein epitopes. The Omicron variant has numerous sequence variations in the spike and nucleocapsid proteins,^19^ which has raised concerns from the US Food and Drug Administration about the analytical and clinical accuracy of rapid antigen–based diagnostic tests.^20^ Therefore, this study assessed the analytical and clinical accuracy of 2 rapid diagnostic tests for detecting SARS-CoV-2 variants (Delta and Omicron) involved with the predominant waves of the COVID-19 pandemic.

## Methods

This diagnostic study assessed the analytical accuracy of 2 rapid antigen tests approved by the US Food and Drug Administration—SCoV-2 Ag *Detect* Rapid Self-Test (InBios International Inc) and BinaxNOW COVID-19 Ag Card (Abbott Laboratories)—using 3 replication-competent variants or strains, including Omicron (B.1.1.529/BA.1), Delta (B.1.617.2), and a wild type of SARS-CoV-2 (USA-WA1/2020). The study received ethical approval from the University of Washington, and participants provided verbal informed consent. The reporting of results followed the Standards for Reporting of Diagnostic Accuracy (STARD) reporting guideline.

The SARS-CoV-2 variants with a known 50% tissue culture infectious dose (TCID_50_) were obtained from BEI Resources. The reported TCID_50_ values for these variants were 4.4 × 10^5^ TCID_50_/mL for Omicron (BA.1), 1.1 × 10^6^ TCID_50_/mL for Delta (B.1.617.2), and 1.6 × 10^6^ TCID_50_/mL for the USA-WA1/2020 strain. The sequences of these variants are available from the Global Initiative on Sharing Avian Influenza Data (BA.1 strain: EPI_ISL_7160424; B.1.617.2 strain: EPI_ISL_2103264) and/or the National Institutes of Health’s GenBank (USA-WA1/2020 strain: MN985325.1). The TCID_50_ value depicts the replication competence of the virus, which was further assessed by measuring the infectious viral particles as plaque-forming units through the infection of VERO E6-ACE2 and TMPRSS2 in a standard plaque assay.^21^

Performance and the analytical limit of detection for the Omicron variant were measured by spiking negative clinical nasal swab matrices with replication-competent virus to establish 3 stock viral concentrations of 5.0 × 10^4^ TCID_50_/mL, 1.25 × 10^4^ TCID_50_/mL, and 3.12 × 10^3^ TCID_50_/mL. From stock concentrations, 20 μL of sample was transferred onto nasal swabs to generate high, medium, and low viral concentrations of 1000 TCID_50_, 250 TCID_50_, and 62.5 TCID_50_ per swab, respectively. Viral dilutions across variants were tested in triplicate for both the SCoV-2 Ag *Detect* Rapid Self-Test and BinaxNOW COVID-19 Ag Card. The results were measured based on the visual signal intensity among serial dilutions.

To complement assessment of the analytical accuracy of the SCoV-2 Ag *Detect* Rapid Self-Test, a clinical diagnostic accuracy study among 802 participants was conducted at multiple testing locations in King County, Washington, from February 17, 2021, to January 11, 2022, during 3 distinct phases of SARS-CoV-2 infection (pre-Delta [February 17 to April 29, 2021], Delta [September 2 to November 30, 2021], and Omicron [December 13, 2021, to January 11, 2022]). The testing periods were determined based on Washington State’s Department of Health data on the circulation of predominant variants.^22^ Participants were aged 18 years or older and reported onset of COVID-19–like symptoms within the prior 5 days. Two anterior nasal swab specimens were collected from each participant—1 for onsite testing by the SCoV-2 Ag *Detect* Rapid Self-Test and 1 for RT-PCR testing. Sex, gender, race, and ethnicity were asked by the research staff and self-reported by the participants.

### Statistical Analysis

Basic descriptive analyses for diagnostic accuracy were conducted, and 95% CIs were computed using the exact binomial (Clopper-Pearson) method. A person was considered COVID-19 positive if they reported COVID-19–like symptoms and had a laboratory-based RT-PCR test positive for SARS-CoV-2. Data were analyzed with R, version 4.2.0 (R Project for Statistical Computing).

## Results

In the clinical study, 802 participants were enrolled (mean [SD] age, 37.3 [13.3] years; 467 [58.2%] female), 424 (52.9%) of whom had not received COVID-19 vaccination. Five of these participants (3 from the Delta phase and 2 from the Omicron phase) were missing RT-PCR results and were excluded from the analyses. Participants were tested a median of 2 days (IQR, 1-3 days) from symptom onset (Table 1). The pre-Delta phase consisted mostly of Alpha, Epsilon, and Gamma variants in this study population. Overall, there were no apparent differences in participants among the 3 study phases, with the exception of increasing rates of vaccination and collection of nasal specimens by a research coordinator during the Delta phase. Across the study period, the median cycle threshold value by RT-PCR among participants positive for SARS-CoV-2 was 26.2 cycles (IQR, 22.9-29.5 cycles).

**Table 1.** Characteristics of Participants Enrolled in the Clinical Accuracy Study

Characteristic	Participants^a^			
	Pre-Delta (n = 296)	Delta (n = 292)	Omicron (n = 214)	All (N = 802)
Sex at birth^b^				
Female	160 (54.1)	191 (65.4)	116 (54.2)	467 (58.2)
Male	135 (45.6)	101 (34.6)	98 (45.8)	334 (41.6)
Gender^b^				
Man	134 (45.3)	103 (35.3)	94 (43.9)	331 (41.3)
Nonbinary or genderqueer	1 (0.3)	5 (1.7)	1 (0.5)	7 (0.9)
Woman	160 (54.1)	184 (63.0)	118 (55.1)	462 (57.6)
Prefer not to answer	0	0	1 (0.5)	1 (0.1)
Race and ethnicity^b^				
American Indian or Alaska Native	6 (2.0)	3 (1.0)	1 (0.5)	10 (1.2)
Asian	27 (9.1)	18 (6.2)	20 (9.3)	65 (8.1)
Black or African American	27 (9.1)	37 (12.7)	23 (10.7)	87 (10.8)
Hispanic or Latinx	52 (17.6)	33 (11.3)	27 (12.6)	112 (14.0)
Native Hawaiian or Pacific Islander	9 (3.0)	12 (4.1)	8 (3.7)	29 (3.6)
White	149 (50.3)	167 (57.2)	116 (54.2)	432 (53.9)
>1	12 (4.1)	19 (6.5)	12 (5.6)	43 (5.4)
Not listed	6 (2.0)	1 (0.3)	1 (0.5)	8 (1.0)
Prefer not to answer	7 (2.4)	2 (0.7)	6 (2.8)	15 (1.9)
Age, mean (SD), y	34.3 (11.0)	38.4 (13.5)	39.8 (15.0)	37.3 (13.3)
Contact with person positive for SARS-CoV-2 infection within prior 2 weeks				
No	182 (61.5)	190 (65.1)	105 (49.1)	477 (59.5)
Yes	75 (25.3)	75 (25.7)	89 (41.6)	239 (29.8)
Unknown	39 (13.2)	27 (9.2)	20 (9.3)	86 (10.7)
Time since symptom onset, median (IQR), d	2 (1-3)	2 (1-3)	2 (1-3)	2 (1-3)
COVID-19 vaccination, doses^c^				
0	296 (100)	95 (32.5)	33 (15.4)	424 (52.9)
1	0	24 (8.2)	12 (5.6)	36 (4.5)
2	0	165 (56.5)	110 (51.4)	275 (34.3)
3	0	7 (2.4)	59 (27.6)	66 (8.2)
Swab sample collection				
All RT-PCR tests				
Supervised self-collection	296 (100)	41 (14.0)	214 (100)	551 (68.7)
Collected by research coordinator	0	251 (86.0)	0	251 (31.3)
RT-PCR positive for SARS-CoV-2				
Supervised self-collection	64 (100)	5 (11.6)	73 (100)	142 (78.9)
Collected by research coordinator	0	38 (88.4)	0	38 (21.1)
Ct values by RT-PCR among specimens positive for SARS-CoV-2, median (IQR)	22.5 (19.6-25.5)	27.1 (24.3-29.9)	27.1 (24.3-29.9)	26.2 (22.9-29.5)

Abbreviations: Ct, cycle threshold; RT-PCR, reverse transcriptase–polymerase chain reaction.

^a^ Data are presented as the number (percentage) of participants unless otherwise indicated.

^b^ Sex assigned at birth, gender, and race and ethnicity were not available for 1 participant from the pre-Delta phase. *Woman* included the responses “woman” and “transgender woman”; *man* included the responses “man” and “transgender man.”

^c^ Vaccination status was not available for 1 participant from the Delta phase.

The diagnostic accuracy of the SCoV-2 Ag *Detect* Rapid Self-Test was consistent across SARS-CoV-2 variants (Table 2). The positive percent agreement ranged from 81.2% (95% CI, 69.5%-89.9%) to 90.7% (95% CI, 77.9%-97.4%). Of importance, the negative percent agreement remained high throughout the study period, with a cumulative diagnostic specificity of 99.8% (95% CI, 99.1%-100%). The assay had improved sensitivity for swab specimens that had a lower cycle threshold by RT-PCR testing, which correlates with a higher viral load (Table 3). Among participants with a cycle threshold of 30 or less, the positive percent agreement was 97.9% (95% CI, 94.1%-99.6%). In addition, there were no apparent differences in clinical test performance when stratified by vaccination status or days since symptom onset.

**Table 2.** Analytical and Clinical Accuracy of SCoV-2 Ag *Detect* Rapid Self-Test Across SARS-CoV-2 Variants^a^

	Pre-Delta^b^	Delta	Omicron	Total^c^
Participants positive for COVID-19/participants with valid RT-PCR results, No. (%)^d^	64/296 (21.6)	43/289 (14.9)	73/212 (34.4)	180/797 (22.6)
Time since symptom onset, mean (SD), d	2.2 (0.2)	2.3 (1.2)	2.5 (1.3)	2.3 (1.2)
Cycle threshold values among specimens positive for SARS-CoV-2 by RT-PCR, mean (SD)	23.9 (5.2)	27.6 (4.6)	28.0 (4.8)	26.5 (5.3)
Agreement for rapid antigen test, % (95% CI)^e^				
Positive	81.2 (69.5-89.9)	90.7 (77.9-97.4)	83.6 (73.0-91.2)	84.4 (78.3-89.4)
Negative	100 (98.4-100)	99.6 (97.8-100)	100 (97.4-100)	99.8 (99.1-100)
Analytical limit of detection for rapid antigen test, TCID_50_ per swab	62.5	62.5	62.5	62.5

Abbreviations: RT-PCR, reverse transcriptase–polymerase chain reaction; TCID_50_, 50% tissue culture infectious dose.

^a^ Testing periods were determined based on Washington State’s Department of Health data on the circulation of predominant variants.^22^ The pre-Delta phase was from February 17 to April 29, 2021; Delta phase, September 2 to November 30, 2021; and Omicron, December 13, 2021, to January 11, 2022.

^b^ The pre-Delta phase consisted mostly of Alpha, Epsilon, and Gamma variants in this study population.

^c^ Five participants (3 from Delta phase, 2 from Omicron phase) were missing RT-PCR results and were excluded from analyses.

^d^ Defined as a person who reported COVID-19–like symptoms and had a laboratory-based RT-PCR test positive for SARS-CoV-2.

^e^ 95% CIs were computed using the exact binomial (Clopper-Pearson) method.

**Table 3.** Clinical Accuracy of SCoV-2 Ag *Detect* Rapid Self-Test Across SARS-CoV-2 Variants by Days Since Symptom Onset, Vaccination Status, and Ct Values by RT-PCR

	Participants, No.^a^	TP/(TP + FN)	PPA (95% CI)^b^	TN/(TN + FP)	NPA (95% CI)^b^
**All phases**					
Overall	797	152/180	84.4 (78.3-89.4)	616/617	99.8 (99.1-100)
Days since symptom onset					
0-3	662	119/141	84.4 (77.3-90.0)	520/521	99.8 (98.9-100)
4-5	135	33/39	84.6 (69.5-94.1)	96/96	100 (96.2-100)
Doses of vaccine					
0	423	77/91	84.6 (75.5-91.3)	332/332	100 (98.9-100)
≥1	373	75/89	84.3 (75.0-91.1)	283/284	99.6 (98.1-100)
Gene Ct value by RT-PCR					
≤33	155	146/155	94.2 (89.3-97.3)	0/0	NC
>33	642	6/25	24.0 (9.4-45.1)	616/617	99.8 (99.1-100)
≤30	145	142/145	97.9 (94.1-99.6)	0/0	NC
>30	652	10/35	28.6 (14.6-46.3)	616/617	99.8 (99.1-100)
**Pre-Delta phase**					
Overall	296	52/64	81.2 (69.5-89.9)	232/232	100 (98.4-100)
Days since symptom onset					
0-3	254	44/53	83.0 (70.2-91.9)	201/201	100 (98.2-100)
4-5	42	8/11	72.7 (39.0-94.0)	31/31	100 (88.8-100)
Vaccine doses					
0	296	52/64	81.2 (69.5-89.9)	232/232	100 (98.4-100)
≥1	0	0/0	NC	0/0	NC
Gene Ct value by RT-PCR					
≤33	58	51/58	87.9 (76.7-95.0)	0/0	NC
>33	238	1/6	16.7 (0.4-64.1)	232/232	100 (98.4-100)
≤30	54	51/54	94.4 (84.6-98.8)	0/0	NC
>30	242	1/10	10.0 (0.3-44.5)	232/232	100 (98.4-100)
**Delta phase**					
Overall	289	39/43	90.7 (77.9-97.4)	245/246	99.6 (97.8-100)
Days since symptom onset					
0-3	244	32/36	88.9 (73.9-96.9)	207/208	99.5 (97.4-100)
4-5	45	7/7	100 (59.0-100)	38/38	100 (90.7-100)
Vaccine doses					
0	94	12/12	100 (73.5-100)	82/82	100 (95.6-100)
≥1	194	27/31	87.1 (70.2-96.4)	162/163	99.4 (96.6-100)
Gene Ct value by RT-PCR					
≤33	38	37/38	97.4 (86.2-99.9)	0/0	NC
>33	251	2/5	40.0 (5.3-85.3)	245/246	99.6 (97.8-100)
≤30	35	35/35	100 (90.0-100)	0/0	NC
>30	254	4/8	50.0 (15.7-84.3)	245/246	99.6 (97.8-100)
**Omicron phase**					
Overall	212	61/73	83.6 (73.0-91.2)	139/139	100 (97.4-100)
Days since symptom onset					
0-3	164	43/52	82.7 (69.7-91.8)	112/112	100 (96.8-100)
4-5	48	8/21	85.7 (63.7-97.0)	27/27	100.0 (87.2-100)
Vaccine doses					
0	33	13/15	86.7 (59.5-98.3)	18/18	100 (81.5-100)
≥1	179	48/58	82.8 (70.6-91.4)	121/121	100 (97.0-100)
Gene Ct value by RT-PCR					
≤33	59	58/59	98.3 (90.9-100)	0/0	NC
>33	153	3/14	21.4 (4.7-50.8)	139/139	100 (97.4-100)
≤30	56	56/56	100 (93.6-100)	0/0	NC
>30	156	5/17	29.4 (10.3-56.0)	139/139	100 (97.4-100)

Abbreviations: Ct, cycle threshold; FN, false negative; FP, false positive; NC, not calculable; NPA, negative percent agreement; PPA, positive percent agreement; RT-PCR, reverse transcriptase–polymerase chain reaction; TN, true negative; TP, true positive.

^a^ Five participants (3 from Delta phase, 2 from Omicron phase) were missing RT-PCR results and were excluded from analyses.

^b^ 95% CIs were computed using the exact binomial (Clopper-Pearson) method.

**Figure 1.**
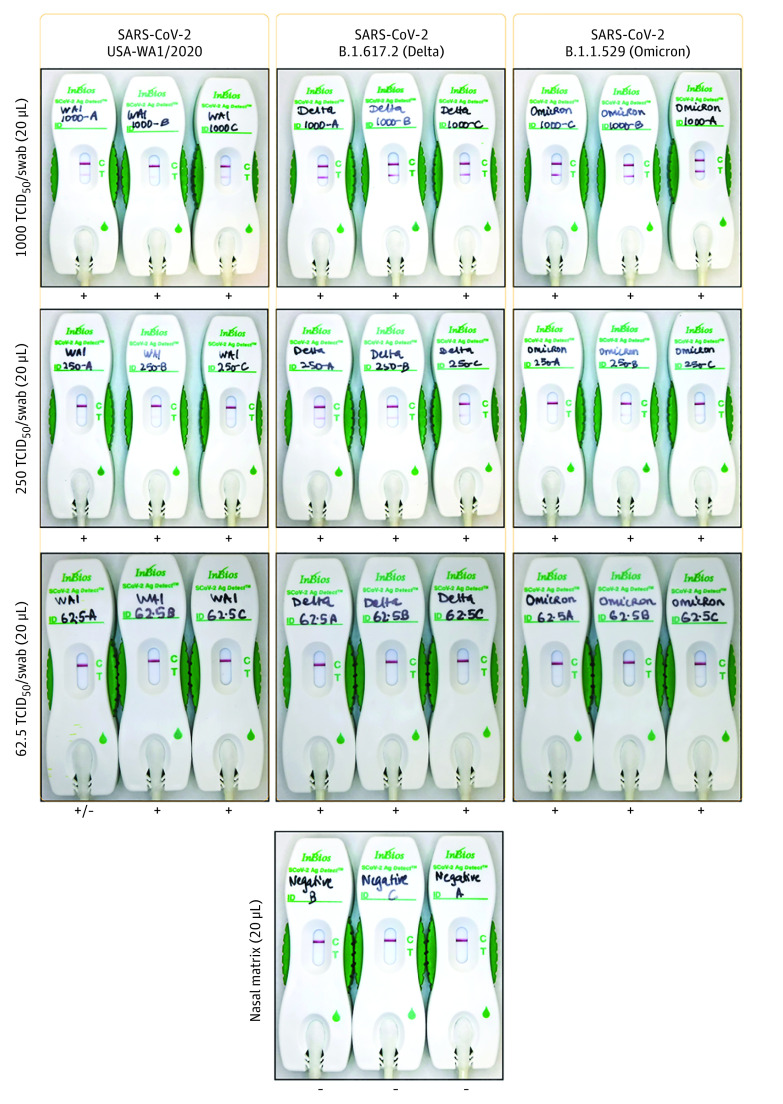
Images of the SCoV-2 Ag *Detect* Rapid Self-Test Across SARS-CoV-2 Variants and Stratified by Viral Load of 50% Tissue Culture Infectious Dose (TCID_50_) per Swab

**Figure 2.**
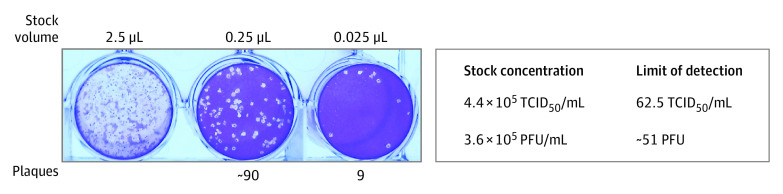
Plaque Assay to Determine the Replication Competence of the SARS-CoV-2 Isolate hCoV-19/USA/MD-HP20874/2021 (Lineage B.1.1.529; Omicron Variant) PFU indicates plaque-forming unit; TCID_50_, 50% tissue culture infectious dose.

In the assessment of analytical accuracy, the visual signals of the SCoV-2 Ag *Detect* Rapid Self-Test were positively associated with viral concentration (Figure 1). The estimated limit of detection for both rapid nucleocapsid antigen tests was at or below 62.5 TCID_50_. In a similar assessment, the dilutions across variants for the BinaxNOW COVID-19 Ag Card were also positively associated with viral concentration, and the estimated limit of detection was consistent across variants. By subsequent plaque assay, which determines the amounts of infectious virus, the limit of detection (62.5 TCID_50_) corresponded to approximately 51 plaque-forming units (Figure 2). For both rapid antigen assays, there were no significant differences in the analytical limit of detection across SARS-CoV-2 variants.

## Discussion

In this diagnostic study, analytical and clinical performance data demonstrated accuracy of 2 rapid antigen tests for detecting SARS-CoV-2 during 3 phases of variants. Other studies have similarly demonstrated good analytical sensitivity for rapid antigen tests to detect Omicron^23,24^ and similar limits of analytical detection between the Omicron variant and the USA-WA1/2020 strain.^25^ A field study showed that the BinaxNOW COVID-19 Ag Card had good clinical accuracy for Omicron.^26^ Overall, this study demonstrated both analytical and clinical accuracy of rapid antigen testing across several variants of concern, including Omicron and Delta.

### Strengths and Limitations

This study has strengths. The detection of circulating SARS-CoV-2 variants was representative of the community prevalence over a 12-month period.

This study also has limitations. It used a 5-day testing window from the onset of symptoms, which is the approved period of use for these rapid diagnostic tests but may not reflect the full spectrum of clinical testing. Additional clinical and analytical comparative studies may be warranted for other rapid antigen tests. The positive and negative predictive values, which are based on the prevalence of disease, may not be generalizable to other populations. In addition, the Omicron strain evaluated was B.1.1.529/BA.1, and the analyses did not include more recent Omicron strains, such as BA.2 or BA.2.12.

## Conclusions

In this diagnostic study, analytical and clinical performance data demonstrated the preserved accuracy of 2 rapid antigen tests across SARS-CoV-2 variants among adults with COVID-19 symptoms. Rapid antigen tests may correlate with the recovery of replication-competent SARS-CoV-2^27^ and appeared to retain accuracy across variants. Although more clinical studies are needed, the ongoing home-based rapid antigen testing programs may be an important intervention to reduce global SARS-CoV-2 transmission.
