# Error in Byline

**DOI:** 10.1001/jamanetworkopen.2022.37825

**Published:** 2022-10-03

In the Original Investigation titled “Accuracy of 2 Rapid Antigen Tests During 3 Phases of SARS-CoV-2 Variants,”^1^ published August 24, 2022, the academic degree shown in the byline for Dr Hau was changed from BA to PhD. This article has been corrected.^1^
